# Cloning, Expression, and Functional Characterization of In-House Prepared Human Leukemia Inhibitory Factor

**Published:** 2013-07-02

**Authors:** Hassan Rassouli, Shiva Nemati, Siamak Rezaeiani, Ali Sayadmanesh, Mohammad Reza Gharaati, Ghasem Hosseini Salekdeh, Hossein Baharvand, Hamid Gourabi

**Affiliations:** 1 Department of Molecular Systems Biology at Cell Science Research Center, Royan Institute for Stem Cell Biology and Technology, ACECR, Tehran, Iran; 2 Department of Stem Cells and Developmental Biology at Cell Science Research Center, Royan Institute for Stem Cell Biology and Technology, ACECR, Tehran, Iran; 3 Department of Genomics, Agricultural Biotechnology Research Institute of Iran, Karaj, Iran; 4 Department of Developmental Biology, University of Science and Culture, ACECR, Tehran, Iran; 5 Department of Genetics at Reproductive Biomedicine Research Center, Royan Institute for Reproductive Biomedicine, ACECR, Tehran, Iran

**Keywords:** Leukemia Inhibitory Factor, Recombinant Protein, Embryonic Stem Cells, Induced Pluripotent Stem Cells, Cell Proliferation

## Abstract

**Objective:**

Leukemia inhibitory factor (LIF) plays important roles in cellular proliferation, growth promotion and differentiation of various types of target cells. In addition, LIF influences bone metabolism, cachexia, neural development, embryogenesis and inflammation. Human LIF (hLIF) is an essential growth factor for the maintenance of mouse embryonic stem cells (ESCs) and induced pluripotent stem cells (iPSCs) in a pluripotent, undifferentiated state.

**Materials and Methods:**

In this experimental study, we cloned hLIF into the pENTR-D/ TOPO entry vector by a TOPO reaction. Next, hLIF was subcloned into the pDEST17 destination vector by the LR reaction, which resulted in the production of a construct that was transferred into *E. coli* strain Rosetta-gami™ 2(DE3) pLacI competent cells to produce the His6-hLIF fusion protein.

**Results:**

This straightforward method produced a biologically active recombinant hLIF protein in *E. coli* that has long-term storage ability. This procedure has provided rapid, cost effective purification of a soluble hLIF protein that is biologically active and functional as measured in mouse ESCs and iPSCs *in vitro*.

**Conclusion:**

Our results showed no significant differences in function between laboratory produced and commercialized hLIF

## Introduction

Leukemia inhibitory factor (LIF) is a pleiotropic glycoprotein that inhibits proliferation of the murine myeloid leukemic cell line M1, while inducing differentiation into macrophages (1). LIF is also known as differentiation-stimulating factor (D-factor) (2), hepatocyte-stimulating factor III (HSF-III) (3), differentiation inhibitory activity (DIA) (4), human interleukin for DA cells (HILDA) (5, 6), melanomaderived lipoprotein lipase inhibitor (MLPLI) (7) and differentiation retarding factor (DRF) (8). Murine and human LIF are highly glycosylated and it has been shown that extensive mannose phosphorylation on LIF plays role in controlling extracellular levels of LIF (9). Its molecular weight varies between 32 to 62 kDa (10, 11), depending on the source. LIF’s biological activity does not appear to be affected in the absence of glycosylation (12, 13). Murine and human LIF are secretory single chain glycoproteins that contain 180 amino acids and three conserved disulfide bonds. There is approximately an 80% amino acid homology between human and mouse LIF (mLIF). mLIF cannot activate human cells whereas human LIF (hLIF) have the capability to activate mouse cells (14).

hLIF plays its roles through a receptor that is comprised of a 190 kDa LIF-binding α-chain and a 130 kDa signal-transducing β-chain (gp130) receptor superfamily. LIF receptors have been identified on the cell membranes of various cells, including ESCs (15, 16), monocytes (17, 18), placental tissue (19) and hepatocytes (20). LIF is an essential growth factor for the maintenance of mouse embryonic stem cells (ESCs) (21) and induced pluripotent stem cells (iPSCs) in a pluripotent and undifferentiated state.

Therefore, this study was initiated to express hLIF *in vitro*. Several groups reported successful production of LIF in *E. coli* (22-24). Here, we have described a straightforward strategy to produce biologically active recombinant hLIF protein in E. coli with long-term storage capabilities. This procedure provides rapid, cost effective purification of soluble hLIF protein that is biologically active and functional. Additionally, this protocol can be used to produce other growth factors.

## Materials and Methods

### Isolation of hLIF cDNA

In this experimental study, Total RNA from human ESCs was isolated using NucleoSpin RNA II (MN, Germany). The first strand of cDNA synthesis was performed using Super Script III reverse transcriptase (Invitrogen, Carlsbad, CA, USA), an oligo dT primer, and 2 μg of purified total RNA. The primers used to amplify hLIF were designed to amplify nucleotides 66-609 (accession no: NM- 002309.3) and exclude the signal peptide coding sequence based according to Genbank. Generated cDNA was amplified with hLIF-topo-F (5' CAC CAG CCC CCT CCC CAT CAC C 3') which introduced a directional cloning site at the 5' end (underlined sequence) and hLIF-R (5' CTG AGA TCC CTC GGT TCA C 3') that included a stop codon for termination of the translation reaction. For fragment amplification, pfx DNA polymerase (Invitrogen, Carlsbad, CA, USA) and a Mastercycler® Gradient PCR (Eppendorf Netheler-Hinz GmbH, Hamburg, Germany) were used. Amplification steps included pre-incubation at 95˚C for 4 minutes; 30 cycles at 95˚C for 30 seconds, 60˚C for 30 seconds, and 68˚C for 40 seconds, followed by one incubation step at 68˚C for 8 minutes. Next, we analyzed the PCR products by electrophoresis on a 1% agarose gel after which they were visualized by ethidium bromide staining under ultra violet (UV) light.

### Construction of the pENTER D-TOPO/hLIF entry clone

The resultant PCR product was cloned into the pENTR-D/TOPO gateway entry vector using the TOPO reaction according to the supplier’s directions (Invitrogen, Carlsbad, CA, USA). The recombinant pENTER D-TOPO/hLIF entry clone was transferred into Library Efficiency® DH5α™ Competent Cells (Invitrogen, Carlsbad, CA, USA) by the heat shock method as described by the manufacturer. Clones were cultured in LB broth overnight and plasmid extraction was performed with the AccuPrep® Plasmid Mini Extraction Kit (Bioneer, Daejeon, Korea). Recombinant vectors were confirmed by PCR using the M13-F and hLIF-R primers which generated an amplicon size of about 700 bp. DNA sequencing of the inserted segment utilizing M13 forward and reverse primers. M13 forward primer (5' GTAAAACGACGGCCAGT 3') and M13 reverse primer (5' AGCGGATAACAATTTCACACAGGA 3') were used in this study.

### Construction of the pDest17/hLIF expression vector

A pENTER D-TOPO/hLIF construct with correct direction and sequence was chosen for the LR reaction in which hLIF was transferred from the entry clone into the pDEST17 destination vector according to the manufacturer’s instructions (Gateway® Technology, Invitrogen, Carlsbad, CA, USA). For disulfide bond formation, the pDEST17/ hLIF expression clone was transferred to *E. coli* strain Rosetta-gami™ 2 (DE3) pLacI Competent Cells (Merck KGaA, Darmstadt, Germany) by the heat shock method as described by the supplier (User Protocol TB009 Rev. F 0104) and recombinant expression vectors confirmed by PCR.

### Recombinant fusion protein expression and purification

Protein expression and purification were performed as previously described (25). Briefly, a clone was grown overnight in LB medium that contained 100 mg/ml ampicillin at 37˚C and shaken at 180 rpm. Next, cultures were diluted 1:100 in fresh LB that contained 100 mg/ml ampicillin and 2% glucose, then cultivated at 37˚C until the OD_600_ of the media reached 0.8. Recombinant fusion protein expression was then induced by the addition of 0.5 mM isopropyl-d-thiogalactopyranoside (IPTG; Fermentas, Vilnius, Lithuania) and cells were grown for 6 hours or longer. Induced cells were harvested by centrifugation at 8000×g for 10 minutes after which the cell pellets were then frozen at -80˚C until protein purification.

Recombinant His6-hLIF fusion protein was purified by the Ni-NTA Fast Start Kit (Qiagen, Valencia, CA, USA) according to the kit’s manual. In each step, 20 μl samples were preserved for further analysis by SDS-PAGE. The purified His6-hLIF fusion proteins was dissolved in storage buffer, sterilized by filteration (0.22 μm), distributed into vials (100 μg/vial), lyophilized, and stored at -80˚C until further use.

### SDS-PAGE, mass spectrometry analysis

We mixed 20 μl of the elution fractions with 5 μl of 5X loading buffer and heated it at 95˚C for 5 minutes. Samples were then analyzed by SDSPAGE on a 12% (w/v) separating gel followed by staining with 0.1% coomassie brilliant blue R-250. Protein bands of interest were excised from the SDS-PAGE gel. Samples were sent for analysis by liquid chromatography coupled with tandem mass spectrometry (LC-MS/MS) to York University.

### Endotoxin testing and Biological assay of RoyanhLIF

The endotoxin test was performed on three samples by using a Pyrogent™ Gel Clot LAL Assay Kit (Lonza, Basel, Switzerland) according to the manufacturer’s instructions. Biological activity of Royan-hLIF was measured by its ability to promote the proliferation of human erythroleukemic cells (TF-1) (26). These cells are known to proliferate in response to LIF as well as GM-CSF, IL-3 and erythropoietin (22). The cell line was purchased from the National cell bank of Iran (NCBI Code: C602). Biological activity was assessed according to Imsoonthornruksa et al. (22) and Kitamura et al. (26) method with minor modifications. TF-l cells were maintained in RPMI 1640 (Invitrogen, 23400-021) supplemented with 10% Fetal bovine serum (FBS) (HyClone, USA, SH30071.03), 100 units/ml penicillin and 100 μg/ml streptomycin (Invitrogen, 15070-063) and recombinant human GM-CSF (1 ng/ml, R&D systems, 215-GM-050). For bioassay of hLIF, the cells were cultured in RPMI 1640 supplemented with 5% FBS for 24 hours without GM-CSF. Serial dilutions (0.37-12 ng/ml) of Royan-hLIF and commercial Chemicon hLIF (Chemicon, LIF1050) were provided in 96- well flat-bottom plate in RPMI with 5% FBS and 1 ng/ml GM-CSF. For the control, the cells were cultured in the absence of hLIF. Subsequently the cells were added in 96-well plate (density of the cells was 5×10^3^ cells/well) and incubated at 37˚C in a humidified, 5% CO_2_ atmosphere. After this time, cells viability was assessed by MTS [3-(4,5-dimethylthiazol- 2-yl)-5-(3-carboxymethoxyphenyl)- 2-(4-sulfophenyl)-2H-tetra zolium] assay. For the MTS assay, the CellTiter 96 aqueous Non-Radioactive cell proliferation assay kit (Promega, USA, G5430) was used. Briefly, 20 μl of the MTS reagent was added into each well and incubated for an additional 3 hours. The absorbance of each well was recorded at 490 nm on Thermo Scientific Microplate Reader. The procedures were repeated at least three times.

### Effects of Royan-hLIF on mouse ESC pluripotency and self-renewal

We expanded the mouse ESC line OG2 as previously described under feeder-free conditions (27, 28). Royan-hLIF in conjunction with commercial hLIF (Merck KGaA, Darmstadt, Germany) was used to compare functionality and efficiency. For this assay we used three groups: i. cells treated with Royan-hLIF (10 ng/ml), ii. cells treated with commercial hLIF (10 ng/ml), and iii. a negative control group that was not treated with hLIF. Mouse ESCs were cultured for 10 passages prior to analysis. Cell morphology, expression of the stemness markers alkaline phosphatase (ALP), Oct4 and SSEA1 in addition to the cell multiplication rate were compared among the different treatments to ensure Royan-hLIF functionality.

### Culturing mouse ESCs

Mouse ESCs were cultured in mouse ESC medium that contained DMEM/F12 (Invitrogen, 21331-020, Carlsbad, CA, USA) supplemented with 15% knockout serum replacement (KOSR, Invitrogen, 10828-028), 2 mM L-glutamine (Invitrogen, 25030-024), 0.1 mM β-mercaptoethanol (Sigma-Aldrich, M7522, Taufkirchen, Germany), 1% nonessential amino acids (Invitrogen, 11140-035), 100 units/ml penicillin and 100 μg/ ml streptomycin (Invitrogen, 15070-063), 10 mM SB431542 (Stemgent, 04-0010, San Diego, CA, USA), 1 μM PD0325901 (Stemgent, 04-0006, San Diego, CA, USA) and 1000 U/ml hLIF (Merck KGaA, Darmstadt, Germany). Cells were grown in 5% CO_2_ at 95% humidity and passaged every seven days. For passaging, cells were washed once with Dulbecco’s phosphate-buffered saline that contained Ca^2+^ and Mg^2+^ (DPBS, Invitrogen, 14040-117), then incubated with DMEM/F12 that contained Trypsin-EDTA at 37˚C for 3 minutes. Cells were collected by gentle pipetting and after neutralization of trypsin with ESC medium; they were replated on gelatin-coated dishes. The medium was changed every day.

### Immunofluorescence and alkaline phosphatase (ALP) staining

Cells were fixed in 4% paraformaldehyde for 20 minutes, then permeabilized with 0.2% triton X-100 for 30 minutes and blocked in 10% goat serum in phosphate-buffered saline (PBS) for 60 minutes. Primary antibody incubation was performed for 1 hour at 37˚C, washed 3 times, and incubated with FITC-conjugated secondary antibody IgG (1:200, Sigma-Aldrich, F9006) as appropriate for 1 hour at 37˚C. Primary antibodies were: anti-OCT-3/4 (1:100, Santa Cruz Biotechnology, SC-5279) and anti-SSEA-1 (1:50, R&D Systems, Minneapolis, MN, USA, MAB2155,) to determine the undifferentiated states of mouse ESCs. For nuclei staining we used DAPI (0.1 μg/ ml, Sigma-Aldrich, D8417). A fluorescent microscope (Olympus, Japan) was used for cell analysis. We performed ALP staining according to the manufacturer’s recommendations.

### MTT assay

mESCs (Please define abbreviation) were proliferated in the presence of commercial and Royan hLIF for 1, 5 and 10 passages. These cells were seeded in 96-well microtitre plate (Corning Cell Wells, Corning, USA). After 48 hours incubation period, 30 μl of 3-(4, 5-dimethyl-2-thiazolyl)-2, 5-diphenyl-2H-tetrazolium bromide (MTT, M5655, Sigma-Aldrich) solution was added to each well, mixed thoroughly via plate shaker for 1 minute, and all plates were again placed in the same incubator. After 4 hours incubation at 37˚C in 5% CO_2_- buffered and humidified incubator, 100 μl dimethyl sulfoxide (DMSO, D2650, Sigma-Aldrich) was added to each well and mixed thoroughly in order to dissolve the formed blue crystals of formazan. All plates were then shaken for 1 minute and incubated for 2 hours. The optical density (OD) of wells was measured by Microplate Reader at 540 nm.

### Cardiac cell differentiation

To evaluate pluripotency of mouse ESC after 10 passages, cells were differentiated into cardiac cells as previously described (29, 30). For mouse ESCc or direct cardiomyocyte differentiation, we cultured 800 cells of mouse ESCs/ 20 μl of ESC medium containing 10-4 M ascorbic acid (Sigma-Aldrich, A4403) in absence of hLIF, for 2 days. ESCs were cultured in hanging drops to produce embryoid bodies (EBs). Subsequently, we cultured the EBs under suspension conditions in bacterial dishes for 5 additional days. On day 7, EBs were separately plated in 1% gelatin-coated wells of a 24-well tissue culture plate for an additional 14 days to allow adherence and development of beating cardiomyocytes.

### Statistical analysis

All results were expressed as mean ± standard deviation (SD). Stemness markers expressions and proliferation assay were analyzed by the t test and p< 0.05 was considered statistically significant.

## Results

**Fig 1:**
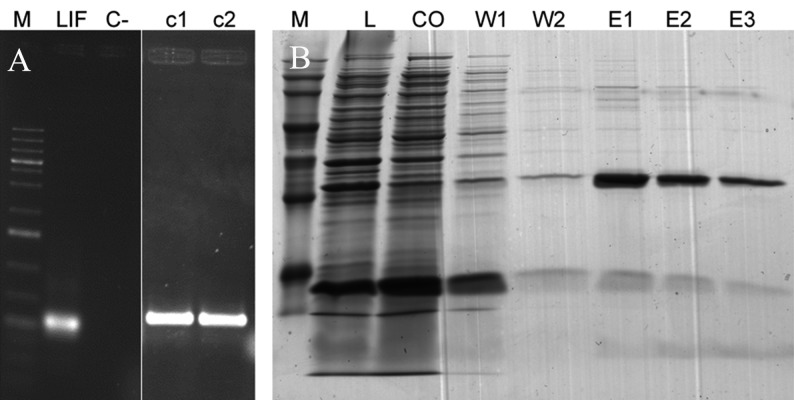
PCR analysis of amplified hLIF and SDS page of produced hLIF. A. The expected 547 bp product of hLIF amplified by PCR with primers that added CCAC to 5' end. M: Size marker; C-: Negative control; LIF: about 700 bp PCR product of hLIF amplified by M13 forward and hLIF reveres primers; c1: Clone 1; c2: Clone 2. B. SDS-PAGE analysis of hLIF production. Recombinant his-tag-hLIF expressed and purified successfully. M: Protein size marker; L: Lysate; CO: Cut off; W1: Wash 1; W2: Wash 2; E1: Elution 1; E2: Elution 2; E3: Elution 3. The purified proteins showed expected size band (21 kD) that represent the target proteins and His-tag). The protein band was excised and analyzed using mass spectrometry that resulted in identification of hLIF as single protein in band.

Cloning of hLIF cDNA and construction of the pENTER D-TOPO/hLIF entry clone The 543 bp hLIF gene was amplified (Fig 1A) from human ESC mRNA and subsequently cloned in a pENTER DTOPO vector to produce a pENTER D-TOPO/hLIF entry clone. The recombinant entry clone was transferred into Library Efficiency® DH5α™ Competent Cells and as a result two clones were appeared next day and both constructs had the correct orientation of the hLIF insertion as shown by PCR analysis. DNA sequencing results showed no mutation in the pENTER D-TOPO/hLIF construct. One of constructs selected for LR reaction randomly.

Construction of the hLIF fusion protein expression vector The selected and confirmed pENTER D-TOPO/hLIF entry clone and pDEST17 destination vector were used for construction of the expression clone with the LR reaction. The pDEST17/hLIF expression clone possesses a T7 promoter, which allows for production of recombinant proteins that contain the N-terminus 6X His-tag. The pDEST17/ hLIF expression clone was transformed into *E. coli* strain Rosetta-gami™ 2(DE3) pLacI (Fig 1B). Out of hundreds of clones, five were tested by colony PCR that utilized T7 forward and hLIF reverse primers. All clones were positive for hILF insertion, which indicated that the LR reaction was 100% efficient.

### Expression and purification of recombinant hLIF fusion protein

*E. coli* strain Rosetta-gami™ 2(DE3) pLacI that contained a pDEST17/hLIF expression clone was grown overnight in LB medium. Cultures were diluted 1:100 in fresh LB and protein expression induced by the addition of IPTG when the OD_600_ of the media reached 0.8. After six hours, the cells were harvested by centrifugation at 8000×g for 10 minutes. Expressed fusion proteins were purified by IMAC on a Nickel 2+ column with 25 mM imidazole, which eliminated the majority of contaminating proteins in the flow through and washing steps. The hLIF fusion protein was obtained in the 250 mM imidazole fractions (Fig 1). The identities of the purified hLIF fusion proteins were confirmed by trypsin digest and LC/MS/MS. The results indicated that our fusion proteins matched hLIF (Accession no.: NM_002309.3; data not shown).

### Endotoxin test and biological activity of RoyanhLIF by MTS assay

**Fig 2:**
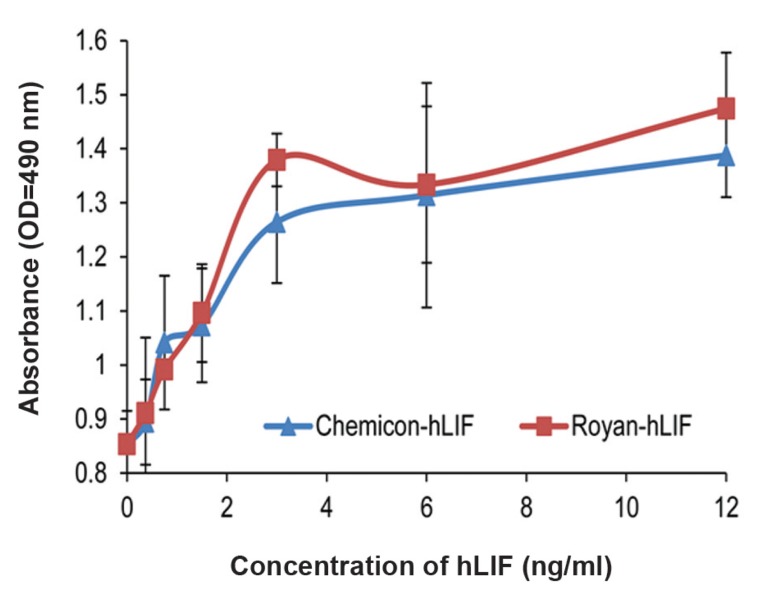
MTS assay of Royan-hLIF and Chemicon hLIF on TF-1 cells by. The proliferation of TF-1 cells in responses to different doses (0.37-12 ng/ml) of recombinant hLIF (■) and commercial Chemicon hLIF (▲) for 72 hours incubation was compared. Results indicate that the Royan-hLIF activity is similar to the commercial hLIF and it can promote the proliferation of TF-1 cells in a dose dependent manner up to 3 ng/ml and there was no significant difference between Royan-hLIF and commercial hLIF.

The endotoxin test showed that all three samples of each hLIF production batch used for the endotoxin detection assay were endotoxin-free. The biological activity of the recombinant hLIF was assessed according to its ability to induce the proliferation in TF-1 cells by MTS assay. The results in figure 2 indicated that the Royan-hLIF can promote the proliferation of TF-1 cells in a dose dependent manner up to 3 ng/ml and its activity is similar to the commercial hLIF.

**Fig 3:**
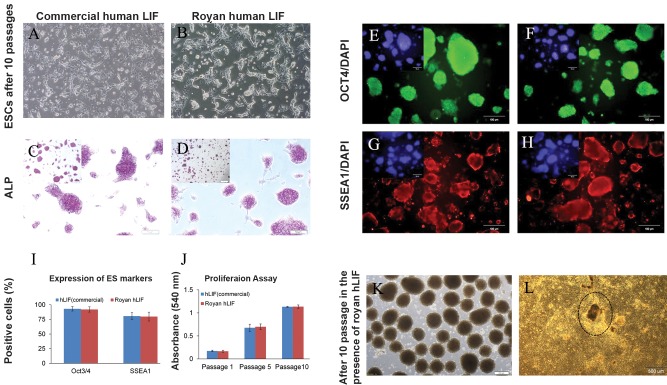
Phase contrast microscopy of OG2 mouse ESC line treated with Royan-hLIF compared to commercial hLIF as the control group, after 10 passages. A. Commercial hLIF (10 ng/ml). B. Royan-hLIF (10 ng/ml). C. Alkaline phosphatase (ALP) staining of the commercial hLIF group. D. ALP staining of Royan-hLIF. E. Immuonofluorescence staining for OCT4 as a pluripotency marker in the commercial hLIF group and F in the Royan-hLIF group. G. Immuonofluorescence staining for SSEA1 as a pluripotency marker in the commercial hLIF group and H in the Royan-hLIF group. I. Quantification of positive cells by immunoflourescence staining. J. MTT assay after 1, 5 and 10 passages of mouse ESCs in the presence of commercial and Royan hLIF. K. Embryoid body (EB) formation for cardiomyocyte differentiation of mouse ESCs after 10 passages using Royan-hLIF. L. Mouse ESC-derived beating colony 5-10 days after differentiation to cardiomyocytes. Results showed that the group treated with 10 ng/ml of Royan hLIF had similar morphological characteristics to the group treated with 10 ng/ml of commercial hLIF. And also mouse ESCs cultured in Royan hLIF for ten passages were induced toward a cardiac cell lineage. According to the results, mouse ESCs cultured with Royan-hLIF efficiently produced beating cells and there was no significant difference between Royan-hLIF and commercial hLIF.

Analysis of the effects of produced hLIF on mouse ESC pluripotency and self-renewal As mentioned above, human and mLIF have an approximately 80% amino acid homology and it has been shown that hLIF can activate mouse cells as well (14). Therefore, we examined the activity of Royan hLIF’s in mouse ESC line during 10 passages. The experiment consisted of Royan-hLIF treated groups, a commercial hLIF treated group and a negative control (without hLIF treatment; Fig 3A, B). All experiments were performed using three biological replicates per treatment. Results showed that the group treated with 10 ng/ml of Royan hLIF had similar morphological characteristics to the group treated with 10 ng/ml of commercial hLIF. The proliferation of mouse ESCs in the presence of commercial and Royan hLIF was assessed by the MTT assay after 1, 5 and 10 passages. The proliferation of cells in Royan hLIF group was as same as commercial hLIF (Fig 3J), with no significant difference observed. The immunostaining for ALP (Fig 3C, D) and pluripotency markers, Oct4 and SSEA1 (Figs 3E-H) confirmed that Royan hLIF was as effective as commercial hLIF. To estimate the proportion of mouse ESC pluripotency, we determined the number of positive cells as a percentage of the total cells in the population following dissociation of the colonies and immunofluorescence staining. We did not observe a significant difference between the two groups in the numbers of positive cells (Fig 3I). Mouse ESCs cultured in Royan hLIF for ten passages were induced toward a cardiac cell lineage. According to the results, mouse ESCs cultured with Royan-hLIF efficiently produced beating cells (Fig 3K, L).

## Discussion

In the present study we cloned a cDNA encoding human LIF into a cloned pENTR-D/TOPO Gateway entry vector using the TOPO reaction, then into a pDEST17 destination vector by using the LR reaction. We have used Gateway Technology because of its rapid, highly efficient technique for cloning and subcloning target genes. This technology provides a wide range of destination vectors for different applications. The pDEST17 vector has a strong promoter which allows for high-level production of recombinant proteins. We used *E. coli* strain Rosetta-gami™ 2 (DE3) pLacI which designed for efficient disulfide band formation. However, the pDEST17/hLIF expression clone has low-to-medium expression of recombinant protein compared with our previous reported recombinant protein, bFGF (25). This might reflect the fact that the hLIF sequence might need codon optimization in order to acquire high protein expression.

The produced recombinant hLIF had correct folding and no inclusion body. Although it has lowto- medium expression levels of recombinant hLIF, its production is still cost-effective due to the low price of LB medium needed to culture the bacteria. To compensate, we could grow additional bacteria. Endotoxins cause serious problems in cell cultures and interfere with other treatments (31, 32). The endotoxin test result for hLIF has shown that all samples were endotoxin-free or their endotoxins, if any, were under detectable levels. This has implied that Royan-hLIF could be used without concern for the presence of endotoxins.

To validate the functionality of hLIF, we applied MTS assy as previously reported by Kitamura et al. (26). We also demonstrated that there was no significant difference between Royan-hLIF and commercial hLIF used in this study. This finding suggests that commercial hLIF can be replaced with Royan-hLIF’s which result in substantial reduction in cost. Mouse ESCs that have been cultured in the presence of Royan-hLIF propagated with a typical round, flat morphology that had definite margins and a high nucleus-cytoplasm ratio. These ESCs expressed ALP and the important markers, Oct4 and SSEA1. Long-term culture of mouse ESCs under these conditions could maintain the differentiation potential of normal cell lines, an important step toward self-sufficiency in large-scale expansion of iPSCs/ESCs.

## Conclusion

Our results indicate that there is not a significant difference in function between in-house generated and commercialized hLIF. The availability of large quantities of recombinant hLIF should greatly facilitate mouse and human pluripotent stem cell cultures.
